# Preliminary impact of the adoption of the Icelandic Prevention Model in Tarragona City, 2015–2019: A repeated cross-sectional study

**DOI:** 10.3389/fpubh.2023.1117857

**Published:** 2023-03-16

**Authors:** Caine C. A. Meyers, Michael J. Mann, Ingibjorg Eva Thorisdottir, Patricia Ros Garcia, Jon Sigfusson, Inga Dora Sigfusdottir, Alfgeir L. Kristjansson

**Affiliations:** ^1^Planet Youth Ltd., Reykjavik, Iceland; ^2^Department of Community and Environmental Health, School of Allied Health Sciences, Boise State University, Boise, ID, United States; ^3^Department of Psychology, Reykjavik University, Reykjavik, Iceland; ^4^Icelandic Centre for Social Research and Analysis (ICSRA), Reykjavik, Iceland; ^5^Department of Social and Behavioral Sciences, School of Public Health, West Virginia University, Morgantown, WV, United States

**Keywords:** Icelandic Prevention Model, primary prevention, adolescents, substance use, Iceland, risk and protective factors, Catalonia, Spain

## Abstract

**Background:**

There is a great need for effective primary prevention intervention strategies to reduce and delay onset of adolescent substance use. The Icelandic Prevention Model (IPM) showed great success in Iceland over the past twenty plus years, however, evidence for the transferability of model is still somewhat limited. Using data collected in Tarragona during regional efforts to begin adoption of the IPM in Catalonia, this study tested the transferability and stability of the core risk and protective factor assumptions of the IPM overtime and examined trends of lifetime smoking, e-cigarette-use, alcohol-use, intoxication, and cannabis-use within the same time period.

**Methods:**

This study includes responses from 15- to 16-years-olds from two region-wide samples taken in 2015 and 2019 in Tarragona (*N* = 2,867). Survey questions assessed frequency of lifetime: smoking, e-cigarette-use, alcohol-use, intoxication, and cannabis-use, and the core model assumptions. Demographic data were also collected. Logistic regression models of main effects with and without time interaction were used to test assumptions and their stability across time. Chi-square tests and Wilcoxon–Mann–Whitney *U* tests were used to compare prevalence of substance use and mean scores of primary prevention variables respectively.

**Results:**

Lifetime: smoking (−7%, *p* < 0.001) and cannabis-use (−4%, *p* < 0.001) decreased, and e-cigarette-use increased (+33%, *p* < 0.001) in Tarragona. Lifetime intoxication (−7%, *p* < 0.001) decreased in a single zone exclusively. Most core model assumptions held in their hypothesised direction across time. The strongest positive association was observed between time spent with parents during weekends and reduced odds of lifetime smoking (OR: 0.62, 95%CI: 0.57–0.67) and the strongest negative association was observed between being outside after midnight and increased odds of lifetime intoxication (OR: 1.41, 95%CI: 1.32–1.51). Mean scores of primary prevention variables also changed disproportionately in Tarragona.

**Conclusion:**

This study confirms that the core IPM assumptions are similar in Tarragona as in Iceland and other contexts previously examined. They also indicate that prevalence of lifetime smoking, intoxication, and cannabis-use decreased disproportionately in Tarragona between 2015 and 2019 during the first phase of regional adoption of the model. Thus, targeting model assumptions represents a viable primary prevention strategy for communities that hope to reduce smoking, alcohol-use, intoxication, and cannabis-use among adolescents.

## Introduction

Alcohol, tobacco, and other drug use (ATOD) by adolescents is a major public health concern worldwide (1). During adolescence, major developmental milestones can be negatively impacted by substance use, which often begins during this life period (2–5). Early onset of substance use can lead to negative short-term and long-term consequences, such as an increased risk of developing substance use disorders (SUDs) later in life (4), disruption of physical and social development (6), and various other negative health outcomes (5).

Recent findings from the European Burden of Disease Study indicate that the impact of alcohol and drug use disorders emerge most notably at age 15, with a significant increase in years lived with disability (YLD) associated with these disorders (5). Additionally, among males aged 20–24, SUDs are among the most common disorders (5). In 2019, the European School Survey Project on Alcohol and Other Drugs (ESPAD) estimated that the average proportion of 15- to-16-year-olds in Europe who start using alcohol before the age of 13 is 33%, with varying rates across countries, ranging from 7.1% in Iceland to 60% in Georgia (3). Similarly, the average proportion of students who reported smoking for the first time at or before 13 years of age is 18%, with varying rates across countries, ranging from 5.4% in Iceland to 33% in Lithuania (3). The large variation in population proportion of early onset substance use between countries highlights a need for the potential of adopting intervention strategies across different countries.

Historically, interventions aimed at preventing drug use among adolescents have shown mixed results. The success of these interventions has varied due to differences in their scope, principles, and implementation methods. For example, strategies, such as those that focus on a zero-tolerance policy towards drug use, scare children away from drug use by exposing them to individuals deemed as criminals, or place responsibility on young people to “just say no,” have been largely unsuccessful in reducing ATOD use and, in some cases, may have even increased the likelihood of substance use (7–9). Effective strategies have been those that emphasise community engagement, family- and school involvement, and positive youth development (10). However, an important unanswered question concerns the somewhat limited evidence of the transferability of such strategies and approaches across different contexts despite their success under the original conditions (11, 12).

### The Icelandic Prevention Model

The Icelandic Prevention Model (IPM) is a primary substance use prevention process tool where the key ingredient is collaboration *via* community engagement, family- and school involvement and pro-social positive youth development. The origins of the model can be traced back to the mid-1990s (13–15) in response to alarmingly high levels of substance use among Icelandic adolescents in the 1995 ESPAD study (16). Since its inception, the IPM has, resulted in significant decreases in adolescent smoking, cannabis-use, alcohol-use, and intoxication over the span of two decades (17–20).

The processes and main axioms that emerged from two decades of this work in Iceland were formalised and published for wider dissemination in 2020 as the IPM's “Five Guiding Principles” and “10 Steps to Implementation” (14, 21). Both documents serve as tools for implementing the IPM to contexts outside of Iceland.

#### The IPM's five guiding principles and 10 steps to implementation

The five guiding principles of the IPM are the main theoretical pillars of the model and are as follows (14):

1. apply a primary prevention approach that is designed to enhance the social environment,
2. emphasise community action and embrace public schools as the natural hub of neighbourhood/area efforts to support child and adolescent health, learning, and life success,
3. engage and empower community members to make practical decisions using local, high-quality, accessible data and diagnostics,
4. integrate researchers, policy makers, practitioners, and community members into a unified team dedicated to solving complex, real-world problems,
5. match the scope of the solution to the scope of the problem, including emphasising long-term intervention and efforts to marshal adequate community resources.

The 10 Steps to Implementation are modelled after two decades of primary prevention work in Iceland that significantly reduced substance use among adolescents. They are serial in nature and can be summarised as follows (21):

1. local coalition identification, development, and capacity building,
2. local funding identification, development, and capacity building,
3. pre-data collection planning and community engagement,
4. data collection and processing, including data-driven diagnostics,
5. enhancing community participation and engagement,
6. dissemination of findings,
7. community goal-setting and other organised responses to the findings,
8. policy and practise alignment,
9. child and adolescent immersion in primary prevention environments, activities, and messages,
10. repeat steps 1–9 annually.

#### IPM risk and protective assumptions and transferability to other contexts

The underlying assumptions of the IPM stem from classical social theories of deviance, which are globally relevant. Those include the Theory of Social Integration (22), Theory of Social Control (23), and Bandura's Social Cognitive Theory (24). In line with the principles of the IPM, the model assumes that adolescents who grow up in supportive environments from their parents or caregivers and family, have non-substance-using friends, attend a supportive and nurturing school, and have access to positive, character-building, and pro-social leisure activities are less likely to engage in substance use at an early age compared to those who lack such support (25–27). As such, the IPM risk and protective factors can be summarised into four key domains: family, peer groups, leisure time, and school. These four domains represent critical measurement factors to be analysed and targeted for relevant policies and intervention practises to reduce the risk of substance use among adolescents. The 10 steps to implementation describe a sequential system where routine survey data is used to guide local public health education processes *via* mass dissemination, local decision making for areas of emphasis, intervention planning and development, and revision and reassessment.

Recently, one study has demonstrated that lifetime alcohol, cannabis, and amphetamine use and intoxication decreased among 10^th^ graders in the three largest cities of Lithuania (Vilnius, Kauna, Klaipeda) following local adoption of the IPM. Additionally, this study showed that the risk and protective factor associations with substance use outcomes were largely the same in Lithuania as in Iceland (28). The IPM is now being adopted to communities worldwide, including places in Europe, North America, Oceania, and Central and Latin America, largely in partnership with the Planet Youth organisation (see https://planetyouth.org). Thus, more evidence for the transferability of the model and its effectiveness is needed.

### Adoption of the Icelandic Prevention Model in Tarragona City

Tarragona City is a municipality situated in the Tarragona province of Catalonia, located on the Mediterranean coast of Spain. The city has an estimated population of around 135,000 individuals (29). Catalonia is an autonomous state within Spain which is linguistically and culturally different from the rest of the country, and considered to be relatively well-off financially. Tourism, manufacturing, and services are among the largest industries in Catalonia, contributing significantly to the local economy (30).

At the time of this study's publication, regional-specific statistics for adolescent substance use were not readily available. However, rates of hospital admissions as a result of substance use were high among adolescents in Tarragona City (31). Additionally, in Spain, in 2015, 78% of children between the ages of 15 to 16 years old reported consuming alcohol, 37% reported smoking at least once in their lifetime, and 27% reported using cannabis (32).

In 2015, the Addictions Prevention Unit of the City of Tarragona received funding from the Strategic Partnership for Youth Erasmus+ initiative to initiative a 2-year pilot to adopt the Icelandic Prevention Model to their local context with support from the Icelandic Centre for Social Research and Analysis (ICSRA). Previously, the Addictions Prevention Unit was working with a communitarian approach to improve the lives and wellbeing of young people, particularly in the context of nightlife, thus the IPM aligned quite well with their goals. The pilot work came to an end in 2017 but efforts to continue implementing the IPM were sustained resulting in two rounds of data collection in the time period between 2015 and 2019.

The preliminary implementation of the IPM in Tarragona City was conducted before the IPM 10-step process had been fully formalised and published, which was not until 2020. The Addictions Prevention Unit of the City of Tarragona collaborated with ICSRA in using the Youth in Europe survey, the predecessor to the Planet Youth survey, to assess risk and protective factors in the four major domains of the IPM, as described above. However, dissemination of the findings and their utilisation varied widely in the different local zones and was not well-documented. To fully understand how the survey data impacted local decision-making and action, a complete process evaluation is needed.

### The purpose of this study

The main purpose of this study was to investigate whether the IPM can be transferred to regions outside of Iceland. To achieve this objective, two waves of cross-sectional data from Catalonia in 2015 and 2019 will be used to examine risk and protective associations embedded with the four key domains of the IPM and substance use outcomes. Secondary analyses will examine trends in substance use and primary prevention variables as related to the model. Because the IPM has shown promising results in reducing substance use outside of Iceland the past (28) and has demonstrated to, be successful in Iceland (17, 18, 20) providing further evidence of its transferability to other contexts will be beneficial for communities worldwide that are seeking strategies to decrease substance use among adolescents, and will address gaps in the somewhat limited evidence for its transferability.

## Materials and methods

### Study sample and data collection

This study used a repeated cross-sectional (pseudo-longitudinal) design, surveying two different cohorts of students in 2015 (*N* = 2,536) and 2019 (*N* = 1,685) to assess changes in adolescent substance use prevalence and associated risk and protective factors in Tarragona, Spain. The surveys were conducted by the Prevention Addiction Unit of Tarragona City Hall in collaboration with the Icelandic Centre for Social Research and Analysis (ICSRA) as part of the Erasmus-funded Youth in Europe project. The Department of Education of the Generalitat of Catalonia gave approval. Surveys were translated to Catalonian and administered in paper form in schools who agreed to participate. A detailed description of the data collection protocol used in this study has been described elsewhere (33).

Schools who did not participate in the survey in both years were excluded from the study, resulting in a loss of three city zones from the final analyses. Only students ages 15- and 16-years-old were included in this study for ease of comparison with other population-based surveys administered in schools such as the ESPAD. The final analyses included 2,867 15- and 16-year-old students across five zones in Tarragona City. Code names were randomly assigned to each zone beginning with “Zone” followed by a number to mitigate undue stigmatisation of areas with differing resources.

### Measures

This study includes variables that are consistent with the theoretical assumptions of the Icelandic Prevention Model (IPM) described in the Five Guiding Principles (14), which are based on established theories of adolescent delinquency and previous research on risk and protective factors related to adolescent substance use (34).

#### Outcome variables

Given the primary prevention nature of the IPM and its environmental focus on cohort differences, the measurement of substance use includes lifetime substance use, including smoking, e-cigarette use, alcohol use, intoxication, and cannabis use. Participants were asked how often they had used these substances in their lifetime, with response options ranging from 0 = “never” to 7 = “forty times or more.” To simplify analyses, responses were grouped as 0 = “never” or 1 = “at least once,” allowing for comparisons of proportions of 2015 and 2019.

#### Independent variables

##### Parental monitoring

Two questions were used to assess parental monitoring, both headed with the title: “How well does the following apply to you?” and the questions: “My parents know whom I am with in the evenings” and “My parents know where I am during the evenings.” Response categories ranged from 1 = “applies very well to me” to 4 = “applies very poorly to me.” Responses were reverse-coded and combined into a scale ranging from 2 to 8 with a higher score indicating greater levels of parental monitoring (α = 0.80).

##### Time spent with parents

Two questions were used to assess Time spent with parents, both headed with the statement: “How well does the following apply to you?” and the questions; “I spend time with my parents during working days” and “I spend time with my parents during the weekends.” Both questions were scored with a range from 1 = “almost never” to 5 = “almost always.” These measures were analysed separately.

##### Intergenerational closure

Two questions were applied to assess Intergenerational Closure, a form of parental social capital. Both questions were headed with the statement: “How well does the following apply to you?” and the statements; “my parents know my friends” and “my parents know the parents of my friends.” Responses ranged from 1 = “applies very well to me” to 4 = “applies very poorly to me” were reverse-coded and combined into one scale ranging from 2 to 8. A higher score indicated greater levels of intergenerational closure (α = 0.73).

##### Peer influence on substance use

Three questions were used to assess peer influence on ATOD use. All were headed with “How well does the following statement apply to you?” and the statements; “sometimes necessary to smoke cigarettes to not be left out of your peer group,” “sometimes necessary to drink alcohol to not be left out of your peer group” and “it is sometimes necessary to use cannabis substances to not be left out of your peer group.” Response categories ranged from 1 = “strongly agree” to 4 = “strongly disagree” and were reverse-coded and combined into a single scale ranging from 3 to 12. Higher scores indicated greater peer influence on substance use (α = 0.84).

##### Peer substance use

Four questions were used to assess peer alcohol, tobacco and other drug use. All questions were headed with “How many of your friends…” and the items; “smoke cigarettes?,” “drink alcohol?,” “get drunk at least once a month?” and “use cannabis substances?” Responses ranged from 1 = “none” to 5 = “almost all” and were combined into a single scale ranging from 4 to 20. This scale was collapsed so that 0 = “none” and 1 = “at least one” (α = 0.90).

##### Negative attitude and motivation towards school

Four questions headed with “How well does the following statement apply to you?” were used to assess attitudes and motivation towards school. Items included “I find school studies pointless,” “I am bored with my studies,” “I am poorly prepared for classes” and “I feel as if I do not put enough effort into my studies.” Responses ranged from 1 = “applies almost always to me” to 5 = “applies almost never to me” and combined into a single scale ranging from 4 to 20. Responses were reverse coded so that higher scores indicate increased negative attitude and motivation towards school (α = 0.72).

##### Outside after midnight

One question was used to assess late outside hours: “During the last seven days, how often were you outside after midnight.” Responses ranged from 1 = “never” to 8 = “7 times.” Due to high positive skew, the responses were dichotomized with 0 = “never” and 1 = “once or more often.”

##### Participation in supervised and organised recreational activities

Two questions were used to assess participation in organised sports and other supervised activities. Those included “How often do you participate in organised sport as part of a club and/or team?” and “How often do you participate in organised recreation or organised extracurricular activities?” Responses ranged from 1 = “almost never” to 6 = “almost every day.”

##### Control variables

Control variables were selected and consistent across all models. Variables were time (0 = 2015, 1 = 2019), gender (0 = male, 1 = female), zone, and relative affluence which was measured with the following question: “how well off is your family compared with other families in your country?” with responses ranging from 1 = much better off to 7 = much worse off.

### Statistical analysis and handling of missing data

Missing data within each individual variable ranged from 0 to 4%, Therefore, no imputations or other handlings of missing data were performed. For all variables, skewness was within ±1.00 and kurtosis within ±2.00.

#### Impact evaluation

This study used logistic regression analyses to (1) assess the transferability of the IPM risk and protective factor assumptions and (2) assess the stability between the risk and protective factor relationships and substance use outcomes at two time points (2015 and 2019).

Two models were selected for the impact evaluation. In model 1, the risk and protective factor and substance use associations were tested on the two cohorts combined without time an interaction. In model 2, time was added as an interaction term. The model equations are as follows:


$$\begin{array}{l} \text{Model 1}\left(\text{M1}\right)\;:\;\text{logit}\left(\text{P}\left(\text{Y}=\text{s}\right)\right)=\text{β}0+\text{β}\text{1}{\;}^{*}\;IPMn+\text{β}\text{2}\\ \;{\;}^{*}\;Time+\text{β}\text{3}{\;}^{*}\;Gender+\text{β}\text{4}{\;}^{*}\;Zone+\text{β}\text{5}\\ \;{\;}^{*}\;Relative\;Affluence\\ \text{Model 2}\left(\text{M2}\right)\;:\;\text{logit}\left(\text{P}\left(\text{Y}=\text{s}\right)\right)=\text{β}0+\text{β}\text{1}{\;}^{*}\;\text{IPMn}+\text{β}\text{2}\\ \;{\;}^{*}\;Time+\text{β}\text{3}{\;}^{*}\;\left(IPMn{\;}^{*}\;Time\right)+\text{β}\text{4}{\;}^{*}\;Year+\text{β}\text{5}\\ \;{\;}^{*}\;Gender+\text{β}\text{6}{\;}^{*}\;Zone+\text{β}\text{7}{\;}^{*}\;Relative\;Affluence \end{array}$$

where, *P* = probability, *s* = substance, *n* = IPM risk or protective factor.

Models 1 and 2 coefficients were exponentiated to calculate odds ratios (OR) and accompanying 95% confidence intervals (CI).

#### Outcome evaluation

In addition to an impact evaluation, this study conducted an outcome evaluation to assess for changes in the proportion of students who had used any of the substances separately in 2015 and 2019. Additionally, changes in the averages of both the risk and protective factors were assessed for in the outcome evaluation. Percentage changes are reported as absolute changes throughout the manuscript.

Proportions of lifetime substance use in 2015 and 2019 were calculated for each zone separately and compared with Chi-squared tests. A grand mean score and individual mean scores were calculated for all zones in 2015 and 2019 and compared using Wilcoxin–Mann–Whitney tests. The significance level for this study was set to 0.05.

## Results

Table 1 shows proportions of lifetime smoking, e-cigarette-use, alcohol-use, intoxication, cannabis-use, and the use of any of e-cigarettes, alcohol, or cannabis in 2015 and 2019 in each of the five Tarragona zones and in all zones combined.

**Table 1:** Comparison of lifetime substance use in Tarragona City using Chi-square tests, 2015–2019.

**Lifetime**	**Z1 (*****N*** = **383)**				**Z2 (*****N*** = **193)**				**Z3 (*****N*** = **499)**			
	**2015**	**2019**	***X** ^2^*	***p***	**2015**	**2019**	***X** ^2^*	***p***	**2015**	**2019**	***X** ^2^*	***p***
Smoking	43	41	0.04	0.83	37	32	0.35	0.55	47	46	0.09	0.76
e-Cigarette	22	56	45.80	< 0.001	9	40	25.54	< 0.001	13	51	88.46	< 0.001
Alcohol	71	73	0.25	0.62	68	63	0.49	0.48	83	78	1.63	0.20
Intoxication	35	42	1.90	0.17	39	38	0.04	0.85	43	46	0.09	0.76
Cannabis	25	27	0.21	0.64	21	24	0.40	0.53	31	27	0.79	0.37
**Lifetime**	**Z4 (*****N*** = **291)**				**Z5 (*****N*** = **1,501)**				**ALL (*****N*** = **2,867)**			
	**2015**	**2019**	***X** ^2^*	***p***	**2015**	**2019**	***X** ^2^*	***p***	**2015**	**2019**	***X** ^2^*	***p***
Smoking	31	27	0.50	0.48	50	40	14.53	< 0.001	46	39	14.26	< 0.001
e-Cig	14	58	56.59	< 0.001	17	46	140.42	< 0.001	16	49	363.47	< 0.001
Alcohol	53	69	7.03	0.008	80	80	0.01	0.92	76	76	0.005	0.94
Intoxication	22	33	0.04	0.85	49	42	5.82	0.02	43	41	1.14	0.29
Cannabis	17	16	0.001	0.98	32	26	5.19	0.02	29	25	4.03	0.04

Table 2 show means of primary prevention variables in 2015 and 2019 in each of the five Tarragona zones and for all zones combined.

**Table 2:** Comparison of mean scores of IPM risk and protective factors in Tarragona City using Wilcoxon–Mann–Whitney *U* tests, 2015–2019.

	**Z1 (*****N*** = **383)**				**Z2 (*****N*** = **193)**				**Z3 (*****N*** = **499)**			
	**2015**	**2019**	***W***	***p***	**2015**	**2019**	***W***	***p***	**2015**	**2019**	***W***	***p***
	**Mean (SD)**	**Mean (SD)**			**Mean (SD)**	**Mean (SD)**			**Mean (SD)**	**Mean (SD)**		
**Family**												
Parental monitoring	7.21 (1.29)	7.12 (1.35)	18,552	0.56	6.87 (1.57)	7.37 (1.17)	3,683.5	0.027	6.84 (1.54)	6.92 (1.49)	28,427	0.56
Time spent with parents during weekdays	3.85 (1.22)	3.00 (1.34)	23,932	< 0.001	3.68 (1.31)	3.32 (1.27)	5,165.5	0.028	3.84 (1.23)	3.20 (1.37)	37,304	< 0.001
Time spent with parents during weekends	3.91 (1.34)	3.79 (1.14)	18,566	0.413	3.89 (1.26)	3.85 (1.28)	4,577	0.673	4.03 (1.20)	3.87 (1.16)	31,885	0.059
Intergenerational closure	6.54 (1.51)	5.46 (1.72)	24,333	< 0.001	5.97 (1.62)	5.36 (1.55)	5,430	0.005	6.00 (1.41)	5.36 (1.55)	36,332	< 0.001
**Peers**												
Peer substance use	9.24 (4.52)	10.07 (4.58)	15,906	0.057	8.90 (4.20)	9.04 (4.31)	4,211.5	0.68	9.68 (4.13)	10.70 (4.60)	25,740	0.022
Peer pressure	3.48 (1.27)	3.45 (1.19)	18,748	0.519	3.62 (1.56)	3.55 (1.59)	4,588.5	0.535	3.66 (1.55)	3.46 (1.31)	31,282	0.042
**School**												
Negative attitude and motivation towards studies	9.62 (3.47)	10.28 (3.40)	15,530	0.025	10.04 (3.14)	10.42 (3.65)	3,985.5	0.496	9.41 (2.98)	10.45 (3.39)	23,711	< 0.001
**Leisure**												
Outside after midnight	1.64 (1.15)	1.94 (1.68)	16,844	0.29	1.59 (1.42)	1.71 (1.48)	4,138	0.328	1.70 (1.30)	2.02 (1.72)	26,525	0.576
Participation in sports	2.52 (1.77)	2.56 (1.84)	17,710	0.828	2.20 (1.66)	2.72 (2.06)	3,845	0.126	2.64 (1.84)	2.77 (1.85)	28,207	0.474
Participation in other supervised activities	2.16 (1.52)	2.63 (1.78)	15,234	0.011	2.12 (1.59)	2.57 (1.69)	3,683	0.043	2.45 (1.71)	2.26 (1.68)	30,768	0.21
	**Z4 (*****N*** = **291)**				**Z5 (*****N*** = **1,501)**				**ALL (*****N*** = **2,867)**			
	**2015**	**2019**	***W***	***p***	**2015**	**2019**	***W***	***p***	**2015**	**2019**	***W***	***p***
	**Mean (SD)**	**Mean (SD)**			**Mean (SD)**	**Mean (SD)**			**Mean (SD)**	**Mean (SD)**		
**Family**												
Parental monitoring	6.82 (1.58)	7.16 (1.42)	9,015.5	0.036	6.67 (1.56)	6.96 (1.36)	239,181	< 0.001	6.79 (1.54)	7.02 (1.38)	901,230	< 0.001
Time spent with parents during weekdays	4.14 (1.13)	3.24 (1.34)	14,285	< 0.001	3.65 (1.38)	3.18 (1.28)	322,295	< 0.001	3.75 (1.32)	3.19 (1.31)	1,231,080	< 0.001
Time spent with parents during weekends	4.18 (1.19)	4.04 (1.07)	11,200	0.071	3.88 (1.22)	3.89 (1.14)	259,458	0.786	3.92 (1.24)	3.89 (1.15)	1,001,014	0.054
Intergenerational closure	5.92 (1.64)	5.55 (1.74)	11,573	0.059	5.83 (1.57)	5.15 (1.73)	317,395	< 0.001	5.96 (1.56)	5.27 (1.71)	1,189,230	< 0.001
**Peers**												
Peer substance use	7.88 (3.93)	8.75 (4.13)	9,064	0.563	10.40 (4.31)	10.15 (4.26)	271,451	0.216	9.81 (4.33)	9.99 (4.39)	952,863	0.292
Peer pressure	3.87 (2.00)	3.61 (1.50)	10,887	0.513	3.74 (1.57)	3.67 (1.42)	272,052	0.344	3.70 (1.58)	3.58 (1.39)	1,024,447	0.021
**School**												
Negative attitude and motivation towards studies	9.41 (2.98)	9.92 (3.44)	8,546.5	0.014	9.82 (3.17)	10.21 (3.27)	241,365	0.009	9.67 (3.16)	9.92 (3.44)	865,907	< 0.001
**Leisure**												
Outside after midnight	1.42 (0.93)	1.91 (1.62)	8,714.5	0.012	1.64 (1.23)	1.85 (1.55)	247,742	0.059	1.63 (1.23)	1.91 (1.62)	906,298	< 0.001
Participation in sports	2.31 (1.72)	2.42 (1.72)	9,835	0.442	2.55 (1.84)	2.91 (1.91)	236,832	< 0.001	2.52 (1.81)	2.76 (1.88)	908,624	< 0.001
Participation in other supervised activities	2.01 (1.50)	2.28 (1.51)	30,768	0.21	2.54 (1.79)	2.82 (1.82)	237,650	0.001	2.40 (1.72)	2.62 (1.76)	900,653	< 0.001

### Substance use and primary prevention variables

#### All zones (Tarragona region)

Prevalence of lifetime: smoking decreased by 7% (*X*^2^ = 14.26, *p* < 0.001) and cannabis-use decreased by 4% (*X*^2^ = 4.03, *p* = 0.04). Prevalence of lifetime e-cigarette-use increased by 33% (*X*^2^ = 363.47, *p* < 0.001). No significant changes in prevalence of lifetime alcohol-use or intoxication were observed.

For protective primary prevention factors, grand means of: parental monitoring increased by 0.23 (*W* = 901,230, *p* < 0.001), time spent with parents during weekends decreased by 0.56 (*W* = 1,231,080, *p* < 0.001), intergenerational closure decreased by 0.69 (*W* = 1,189,230, *p* < 0.001), participation in sports increased by 0.24 (*W* = 908,624, *p* < 0.001), and participation in organised recreation increased by 0.22 (*W* = 900,653, *p* < 0.001) points. No change in time spent with parents during weekends was observed.

For risk primary prevention factors, grand means of: negative attitude and motivation towards studies increased (*W* = 865,907, *p* < 0.001) and being outside after midnight increased (*W* = 906,298, *p* < 0.001). No other significant changes were observed.

#### Zone 1

Prevalence of lifetime e-cigarette use increased by 23% (*X*^2^ = 45.80, *p* < 0.001). No significant changes in prevalence of lifetime smoking, alcohol-use, intoxication, or cannabis-use were observed.

For protective primary prevention factors, mean scores of: time spent with parents during weekdays deceased by 0.85 (*W* = 23,932, *p* < 0.001), intergenerational closure decreased by 1.08 (*W* = 24,333, *p* < 0.001), and participation in organised recreation increased by 0.47 (*W* = 15,234, *p* = 0.011). No other changes in primary protective factors were observed.

For primary prevention risk factors, mean scores of negative attitude and motivation towards studies increased by 0.66 (*W* = 15,530, *p* = 0.025). No other changes in mean risk factor scores were observed.

#### Zone 2

Prevalence of lifetime e-cigarette use increased by 31% (*X*^2^ = 25.54, *p* < 0.001). No significant changes in prevalence of lifetime smoking, alcohol-use, intoxication, or cannabis-use were observed.

For protective primary prevention factors, mean scores of: parental monitoring increased by 0.50 (*W* = 3,683.5, *p* = 0.027), time spent with parents during weekdays decreased by 0.33 (*W* = 5,165.5, *p* = 0.028), intergenerational closure decreased by 0.62 (*W* = 5,430, *p* = 0.005), and participation in organised recreation increased by 0.45 (*W* = 3,683, *p* = 0.043). No other changes in protective factors were observed.

For primary prevention risk factors, no changes in mean scores were observed.

#### Zone 3

Prevalence of lifetime e-cigarette-use increased by 38% (*X*^2^ = 88.46, *p* < 0.001). No significant changes in prevalence of lifetime smoking, alcohol-use, intoxication, or cannabis-use were observed.

For primary prevention factors, mean scores of: time spent with parents during weekdays decreased by 0.64 (*W* = 37,304, *p* < 0.001) and intergenerational closure decreased by 0.64 (*W* = 36,332, *p* < 0.001). No other significant change in prevention factors were observed.

For risk primary prevention factors, mean scores of: peer substance use increased by 1.02 (*W* = 25,740, *p* = 0.022), peer pressure on substance use increased by 0.24 points (*W* = 26,741, *p* = 0.042), and negative attitude and motivation towards studies increased by 1.04 (*W* = 23,711, *p* < 0.001). No other significant changes in risk factor mean scores were observed.

#### Zone 4

Prevalence of lifetime e-cigarette-use increased by 44% (*X*^2^ = 14.53, *p* < 0.001). Prevalence of lifetime alcohol-use increased by 16%. No significant changes were observed in lifetime cigarette-use, intoxication, or cannabis-use.

For protective primary prevention factors, mean score of: parental monitoring increased by 0.34 (*W* = 9,015.5, *p* = 0.036) and time spent with parents during weekdays decreased by 0.90 (*W* = 14,285, *p* < 0.001). No other significant changes in protective factors were observed.

For primary prevention risk factors, mean scores of: negative attitude and motivation towards studies increased by 0.51 (*W* = 8,546.5, *p* = 0.014) and being outside after midnight increased by 0.49 (*W* = 8,714.5, *p* = 0.012). No other significant changes in risk factors were observed.

#### Zone 5

Prevalence of absolute percentage of lifetime: smoking decreased by 10% (*X*^2^ = 14.53, *p* < 0.001), intoxication decreased by 7% (*X*^2^ = 5.82, *p* = 0.02), and cannabis-use decreased by 6% (*X*^2^ = 5.19, *p* = 0.002). Conversely, prevalence of lifetime e-cigarette-use increased by 29% (*X*^2^ = 140.42, *p* < 0.001). No changes were observed in prevalence of lifetime alcohol-use.

For primary prevention protective factors, mean scores of: parental monitoring increased by 0.29 (*W* = 239,181, *p* < 0.001), time spent with parents during weekdays decreased by 0.47 (*W* = 322,295, *p* < 0.001), intergenerational closure decreased by 0.68 (*W* = 317,395, *p* < 0.001), participation in sports increased by 0.36 (*W* = 236,832, *p* < 0.001) and participation in organised recreation increased by 0.28 (*W* = 237,650, *p* = 0.001). No other significant changes in protective factors were observed.

For risk factors, mean scores of negative attitude and motivation towards studies increased by 0.39 (*W* = 241,365, *p* = 0.009). No other significant changes in mean risk factor scores were observed.

#### Risk and protective factor associations

Table 3 contains the logistic regression results from both the main-effects model and the main-effects model with time added as an interaction. Results are presented as odds with accompanying 95% confidence intervals.

**Table 3:** Exponentiated logistic regression results and accompanying 95% confidence intervals of IPM risk and protective factors and lifetime substance use.

	**Smoking**	**E-Cigarettes**	**Alcohol**	**Intoxication**	**Cannabis**
	**OR (95% CI)**	**OR (95% CI)**	**OR (95% CI)**	**OR (95% CI)**	**OR (95% CI)**
**Family**					
**Model 1**					
Parental monitoring	0.68 (0.64–0.72)	0.73 (0.69–0.77)	0.79 (0.74–0.85)	0.70 (0.66–0.74)	0.71 (0.67–0.75)
Time	0.83 (0.70–0.97)	6.11 (5.06–7.39)	1.10 (0.92–1.33)	1.03 (0.88–1.21)	0.95 (0.79–1.13)
**Model 2**					
Parental monitoring	0.70 (0.65–0.75)	0.76 (0.70–0.82)	0.86 (0.80–0.94)	0.75 (0.70–0.80)	0.74 (0.69–0.79)
Parental monitoring^*^time	0.90 (0.80–1.02)	0.89 (0.79–1.01)	0.75 (0.64–0.88)	0.82 (0.73–0.93)	0.9 (0.80–1.01)
**Model 1**					
Time spent with parents on weekdays	0.80 (0.76–0.85)	0.83 (0.77–0.88)	0.81 (0.75–0.87)	0.77 (0.73–0.82)	0.80 (0.75–0.85)
Time	0.68 (0.58–0.80)	4.84 (4.04–5.81)	0.93 (0.77–1.12)	0.83 (0.70–0.97)	0.77 (0.64–0.92)
**Model 2**					
Time spent with parents on weekdays	0.81 (0.75–0.87)	0.85 (0.77–0.94)	0.82 (0.75–0.90)	0.77 (0.72–0.83)	0.80 (0.74–0.86)
Time spent with parents on weekdays^*^time	0.98 (0.87–1.10)	0.95 (0.83–1.08)	0.96 (0.83–1.11)	1.00 (0.89–1.13)	1.00 (0.88–1.14)
**Model 1**					
Time spent with parents on weekends	0.63 (0.59–0.68)	0.69 (0.64–0.74)	0.74 (0.68–0.80)	0.65 (0.61–0.70)	0.63 (0.59–0.67)
Time	0.75 (0.64–0.89)	5.66 (4.69–6.82)	1.02 (0.84–1.22)	0.93 (0.79–1.09)	0.86 (0.72–1.03)
**Model 2**					
Time spent with parents on weekends	0.62 (0.57–0.67)	0.70 (0.64–0.78)	0.75 (0.68–0.84)	0.65 (0.59–0.70)	0.63 (0.58–0.69)
Time spent with parents on weekends^*^time	1.06 (0.93–1.22)	0.96 (0.82–1.11)	0.94 (0.79–1.12)	1.02 (0.89–1.17)	1.00 (0.86–1.15)
**Model l**					
Intergenerational closure	0.82 (0.78–0.86)	0.87 (0.82–0.92)	0.91 (0.86–0.96)	0.85 (0.81–0.89)	0.83 (0.79–0.88)
Time	0.68 (0.57–0.80)	4.96 (4.13–5.96)	1.01 (0.84–1.22)	0.86 (0.73–1.01)	0.77 (0.64–0.92)
**Model 2**					
Intergenerational closure	0.82 (0.77–0.88)	0.82 (0.75–0.89)	0.94 (0.88–1.02)	0.83 (0.77–0.88)	0.83 (0.77–0.89)
Intergenerational closure^*^time	0.99 (0.90–1.10)	1.11 (1.00–1.24)	0.91 (0.81–1.02)	1.06 (0.96–1.17)	1.02 (0.92–1.13)
**Peers**					
**Model 1**					
Peer substance use	1.25 (1.23–1.28)	1.20 (1.17–1.22)	1.25 (1.22–1.28)	1.29 (1.26–1.32)	1.33 (1.29–1.36)
Time	0.70 (0.59–0.84)	6.27 (5.16–7.63)	1.00 (0.82–1.21)	0.89 (0.74–1.06)	0.79 (0.65–0.96)
**Model 2**					
Peer substance use	1.24 (1.21–1.28)	1.16 (1.12–1.19)	1.23 (1.19–1.28)	1.27 (1.23–1.31)	1.31 (1.27–1.35)
Peer substance use^*^time	1.02 (0.97–1.06)	1.08 (1.03–1.13)	1.03 (0.98–1.09)	1.05 (1.00–1.10)	1.04 (0.99–1.10)
**Model 1**					
Peer pressure	1.44 (1.35–1.53)	1.21 (1.14–1.28)	1.36 (1.24–1.49)	1.37 (1.29–1.46)	1.28 (1.22–1.35)
Time	0.79 (0.67–0.92)	5.52 (4.59–6.63)	1.07 (0.89–1.29)	0.98 (0.84–1.15)	0.89 (0.75–1.06)
**Model 2**					
Peer pressure	1.46 (1.34–1.59)	1.19 (1.11–1.28)	1.38 (1.23–1.55)	1.35 (1.26–1.46)	1.27 (1.19–1.35)
Peer pressure^*^time	0.96 (0.84–1.09)	1.04 (0.92–1.17)	0.97 (0.80–1.17)	1.03 (0.91–1.18)	1.04 (0.90–1.16)
**School**					
**Model 1**					
Attitude and motivation towards studies	1.16 (1.13–1.19)	1.10 (1.07–1.13)	1.04 (1.01–1.07)	1.11 (1.08–1.13)	1.14 (1.11–1.17)
Time	0.70 (0.59–0.82)	5.07 (4.23–6.08)	1.03 (0.86–1.24)	0.89 (0.76–1.05)	0.79 (0.66–0.95)
**Model 2**					
Negative attitude and motivation towards studies	1.17 (1.13–1.21)	1.19 (1.11–1.28)	1.04 (1.00–1.08)	1.10 (1.07–1.14)	1.16 (1.12–1.20)
Negative attitude and motivation towards studies^*^time	0.97 (0.92–1.02)	1.04 (0.92–1.17)	1.00 (0.94–1.06)	1.01 (0.96–1.07)	0.96 (0.91–1.01)
**Leisure time**					
**Model 1**					
Outside after midnight	1.37 (1.28–1.46)	1.23 (1.16–1.30)	1.31 (1.20–1.43)	1.41 (1.32–1.51)	1.31 (1.23–1.38)
Time	0.70 (0.60–0.82)	5.07 (4.22–6.08)	0.98 (0.82–1.18)	0.87 (0.74–1.02)	0.80 (0.67–0.96)
**Model 2**					
Outside after midnight	1.44 (1.30–1.59)	1.18 (1.08–1.29)	1.25 (1.10–1.41)	1.45 (1.32–1.61)	1.41 (1.29–1.54)
Outside after midnight^*^time	0.92 (0.81–1.04)	1.08 (0.96–1.23)	1.11 (0.92–1.33)	0.94 (0.83–1.08)	0.87 (0.77–0.98)
**Model 1**					
Participation in sports	1.00 (0.96–1.04)	1.04 (0.99–1.09)	1.07 (1.02–1.13)	1.06 (1.02–1.11)	1.02 (0.97–1.07)
Time	0.77 (0.65–0.90)	5.13 (4.28–6.14)	1.02 (0.85–1.22)	0.93 (0.79–1.09)	0.85 (0.72–1.02)
**Model 2**					
Participation in sports	0.98 (0.93–1.04)	1.00 (0.93–1.07)	1.05 (0.98–1.12)	1.04 (0.98–1.10)	1.00 (0.94–1.01)
Participation in sports^*^time	1.04 (0.96–1.13)	1.08 (0.99–1.19)	1.06 (0.96–1.17)	1.05 (0.97–1.15)	1.05 (0.96–1.15)
**Model 1**					
Participation in other supervised activities	0.93 (0.89–0.97)	1.00 (0.95–1.05)	1.04 (0.99–1.10)	0.99 (0.95–1.04)	0.96 (0.92–1.01)
Time	0.78 (0.66–0.91)	5.18 (4.33–6.21)	1.03 (0.86–1.24)	0.95 (0.81–1.12)	0.88 (0.74–1.05)
**Model 2**					
Participation in other supervised activities	0.9 (0.85–0.95)	0.96 (0.89–1.04)	1.00 (0.94–1.07)	0.99 (0.94–1.05)	0.91 (0.85–0.97)
Participation in other supervised activities^*^time	1.09 (0.99–1.19)	1.07 (0.96–1.18)	1.09 (0.98–1.21)	1.00 (0.92–1.10)	1.15 (1.04–1.27)

### Protective factors

In model 1, parental monitoring was associated with decreased odds of lifetime: smoking (OR: 0.68, 95%CI: 0.64–0.72), e-cigarette-use (OR: 0.73, 95%CI: 0.69–0.77), alcohol-use (OR: 0.79, 95%CI: 0.74–0.85), intoxication (OR: 0.70, 0.66–0.74) and cannabis-use (OR: 0.71, 95%CI: 0.67–0.75). Time was associated with decreased odds in lifetime smoking (OR: 0.83, 95%CI: 0.70–0.97) and an increased odds in lifetime e-cigarette-use (OR: 6.11, 95%CI: 5.06–7.39). In model 2, parental monitoring with time added as an interaction was associated with decreased odds of lifetime: alcohol-use (OR: 0.75, 95%CI: 0.64–0.88) and intoxication (OR: 0.82, 95%CI: 0.73–0.93). No other significant time interactions were observed.

In model 1, time spent with parents during weekdays was associated with decreased odds of lifetime: smoking (OR: 0.80, 95%CI: 0.76–0.85), e-cigarette-use (OR: 0.83, 95%CI: 0.77–0.88), alcohol-use (OR: 0.81, 95%CI: 0.75–0.87), intoxication (OR: 0.77, 95%CI: 0.73–0.82), cannabis-use (OR: 0.80, 95%CI: 0.75–0.85). Time was associated with decreased odds in lifetime: smoking (OR: 0.68, 95%CI: 0.58–0.80) and intoxication (OR: 0.83, 95%CI: 0.70–0.97), and with increased odds of lifetime e-cigarette-use (OR: 4.84, 95%CI: 4.04–5.81). In model 2, no significant time interactions were observed.

In model 1, time spent with parents during weekends was associated with decreased odds of lifetime: smoking (OR: 0.63, 95%CI: 0.59–0.68), e-cigarette-use (OR: 0.69, 95%CI: 0.64–0.74), alcohol-use (OR: 0.74, 95%CI: 0.68–0.80), intoxication (OR: 0.65, 95%CI: 0.61–0.70), and cannabis-use (OR: 0.63, 95%CI: 0.59–0.67). Time was associated with decreased odds of lifetime smoking (OR: 0.75, 95%CI: 0.64–0.89) and increased odds of lifetime e-cigarette-use (OR: 5.66, 95%CI: 4.69–6.82). In model 2, no significant time interactions were observed.

In model 1, intergenerational closure was associated with decreased odds of lifetime: smoking (OR: 0.82, 95%CI: 0.78–0.86), e-cigarette-use (OR: 0.87, 95%CI: 0.82–0.92), alcohol-use (OR: 0.91, 95%CI: 0.86–0.96), intoxication (OR: 0.85, 95%CI: 0.81–0.89), and cannabis-use (OR: 0.83, 95%CI: 0.79–0.88). Time was associated with associated with decreased odds of lifetime: smoking (OR: 0.68, 95%CI: 0.57–0.80) and cannabis-use (OR: 0.77, 95%CI: 0.64–0.92), and increased odds in lifetime e-cigarette-use (OR: 4.96, 95%CI: 4.13–5.96). In model 2, intergenerational closure was associated with increased odds of lifetime e-cigarette-use (OR: 1.11, 95%CI: 1.00–1.24). No other significant associations in model 2 were observed.

In model 1, no significant interactions between sports participation and any of the lifetime substance use outcomes were observed. Time was associated with decreased odds in lifetime smoking (OR: 0.77, 95%CI: 0.65–0.90) and increased odds in lifetime e-cigarette-use (OR: 5.13, 95%CI: 4.28–6.14). Similarly, in model 2, no significant time interactions were observed.

In mode 1, participation in organised recreation was associated with decreased odds in lifetime smoking (OR: 0.93, 95%CI: 0.89–0.97). Time was associated with decreased odds in lifetime smoking (OR: 0.78, 95%CI: 0.66–0.91). No other significant reactions in this model were observed. In model 2, participation in organised recreation with time added as an interaction was associated with increased odds in lifetime cannabis-use (OR: 1.15, 95%CI: 1.04–1.27). No other significant associations in model 2 were observed.

### Risk factors

In model 1, peer substance use was associated with increased odds of lifetime: smoking (OR: 1.25, 95%CI: 1.23–1.28), e-cigarette-use (OR: 1.20, 95%CI: 1.17–1.22), alcohol-use (OR: 1.25, 95%CI: 1.22–1.28), intoxication (OR: 1.29, 95%CI: 1.26–1.32), and cannabis-use (OR: 1.33, 95%CI: 1.29–1.36). Time was associated with decreased odds of lifetime smoking (OR: 0.70, 95%CI: 0.59–0.84) and cannabis-use (OR: 0.79, 95%CI: 0.65–0.96). In model 2, peer substance use with time added as an interaction was associated with increased odds of e-cigarette-use (OR: 1.08, 95%CI: 1.03–1.13) and intoxication (OR: 1.05, 95%CI: 1.00–1.10). No other significant time interactions in model 2 were observed.

In model 1, peer pressure was associated with increased odds of lifetime: smoking (OR: 1.44, 95%CI: 1.35–1.53), e-cigarette-use (OR: 1.24, 95%CI: 1.14–1.28), alcohol-use (OR: 1.36, 95%CI: 1.24–1.49), intoxication (OR: 1.37, 95%CI: 1.29–1.46), and cannabis-use (OR: 1.28, 95%CI: 1.22–1.35). Time was associated with decreased odds lifetime: smoking (OR: 0.79, 95%CI: 0.67–0.92) and increased odds of lifetime e-cigarette-use (OR: 5.52, 95%CI: 4.59–6.63). In model 2, no significant time interactions were observed.

In model 1, negative attitude and motivation towards studies was associated with increased odds of lifetime: smoking (OR: 1.17, 95%CI: 1.13–1.21), e-cigarette-use (OR: 1.19, 95%CI: 1.11–1.28), intoxication (OR: 1.10, 95%CI: 1.07–1.14), and cannabis-use (OR: 1.16, 95%CI: 1.12–1.20). Time was associated with decreased odds lifetime: smoking (OR: 0.70, 95%CI: 0.59–0.82) and cannabis-use (OR: 0.79, 95%CI: 0.66–0.95). In model 2, no significant time interactions were observed.

In model 1, being outside after midnight was associated with increased odds of lifetime: smoking (OR: 1.37, 95%CI: 1.28–1.46), e-cigarette-use (OR: 1.23, 95%CI: 1.16–1.30), alcohol-use (OR: 1.31, 95%CI: 1.20–1.43), intoxication (OR: 1.41, 95%CI: 1.32–1.51), and cannabis-use (OR: 1.31, 95%CI: 1.23–1.38). Time was associated with decreased odds of smoking (OR: 0.70, 95%CI: 0.60–0.82) and cannabis-use (OR: 0.80, 95%CI: 0.67–0.96), and increased odds of lifetime e-cigarette-use (OR: 5.07, 95%CI: 4.22–6.08). In model 2, being outside after midnight with time added as an interaction was associated with decreased odds in lifetime cannabis-use (OR: 0.87, 95%CI: 0.77–0.98). No other significant time interactions were observed.

## Discussion

### Transferability of the IPM

This study confirms that the IPM assumptions regarding risk and protective factors and substance use outcomes apply to the context of Tarragona City and are relatively stable over time, with a few notable exceptions. Like Iceland, factors embedded within the four key domains of the model, such as parental monitoring, spending time with parents, and intergenerational closure were associated with decreased odds of smoking, e-cigarette-use, alcohol-use, intoxication, and cannabis-use. On the other hand, negative attitude and motivation towards studies, peer pressure, peer substance use, and being outside after midnight were associated with increased odds of using the same substances.

Neither participation in sports nor in other supervised activities were significantly related to the odds of lifetime smoking or e-cigarette-use. However, participation in other supervised activities was found to be associated with increased odds of cannabis-use over time. In addition, participation in sports was found to be associated with increased odds of alcohol use and intoxication, and this association remained stable over time. These findings are consistent with those found in other studies (28, 34) and suggest that the context and organisation of extracurricular activities may be more important for substance use prevention than the activities themselves. Communities who wish to increase youth participation in recreational activities in order to decrease the likelihood of substance use should carefully consider the quality and duration of programming along with sufficient levels of supervision and the role of mentoring (26, 27).

### Trends in prevalence of substance use, primary prevention variables

In conjunction with findings from the preliminary impact evaluation, results from the outcome evaluation suggest that the implementation of the IPM may have contributed to declines in lifetime prevalence of smoking behaviour, cannabis-use, and intoxication, particularly in Zone 5 of Tarragona City.

In 2019, Zone 5 had a decrease in lifetime smoking, intoxication, and cannabis-use, but an increase in lifetime e-cigarette use. This trend of increased e-cigarette-use was observed in all zones, with the highest increase occurring in Zone 4 and the smallest increase in Zone 5. For all risk and protective factors, time was independently associated with an increase in e-cigarette-use. Increases in proportions of e-cigarette-use are consistent with what has been found elsewhere in a similar time-period, including in Iceland, although the most recent data from Iceland suggest a considerable regression to a decline in e-cigarette use (35).

Despite the increase in e-cigarette use from 2015 to 2019, the use of e-cigarettes in Zone 5 was still lower than the baseline lifetime smoking rate in 2015, which may be particularly noteworthy. Additionally, the prevalence of lifetime smoking decreased in Zone 5, while it remained unchanged in the other four zones, despite a national increase in Spain from 2015 to 2019. For all risk and protective factors, time was associated with decreased odds of lifetime smoking. Studies that have previously examined trends of e-cigarette-use have reported little to no change in cigarette-use despite increases in e-cigarette-use (36, 37). Thus, these findings seem to suggest that smoking behaviour in Zone 5 has decreased overall.

Decreases in smoking behaviour, cannabis-use, and intoxication in Zone 5 may be due to increased parental monitoring and a general reduction in substance use among adolescents. Parental monitoring has consistently been linked to reduced substance use in adolescents (20, 25, 34), and in this study, it was associated with lower odds of intoxication and alcohol use. Time did not show an independent effect, but parental monitoring was associated with a greater decrease in alcohol use over time in model 2.

Conversely, peer substance use has been linked to increased substance use among adolescents in the past (27, 34), and in this study, its association with e-cigarette-use and intoxication weakened over time. These findings may be reflective of changes, such as declines in overall substance use by peers, or changes in the nature of the relationship between peer substance use and adolescent e-cigarette-use; possibly impacted by the primary prevention efforts carried out in this area.

Regarding cannabis-use, its association with going outside after midnight also weakened over time. Going outside after midnight is a well-established risk factor of adolescent substance use as it increases the likelihood of young people encountering situations where substances are being used, notably by peers (34). This may be further evidenced by increases in lifetime alcohol-use in Zone 4, the only zone where mean scores of going outside after midnight had significantly increased. While mean scores of going outside after midnight did not change in Zone 5, these results may be reflective of changes to the outside environment, such as decreased access and availability of cannabis; again possibly due to primary prevention efforts. Time was independently associated with decreased odds of lifetime cannabis-use in model 1 for peer substance use. Thereby, potentially further suggesting declines in overall peer substance use.

Although increases in parental monitoring were observed in all zones but one, only Zone 5 had a decrease in substance use. This may be due to differences in population characteristics and resource availability across zones. In this study, Zone 5 represents the most central and populated zone of the city. Step 9 of the IPM Steps to Implementation requires adolescents to be immersed in the primary prevention environment (i.e., where they spend most of their time) and it is widely known that resource availability and attention can vary across different areas in population-based interventions (38). To better understand and explain these differences in Tarragona City, future studies, including a full process evaluation, are recommended. Similarly, a full process evaluation will provide insight into which risk and protective factor domains were emphasised in the primary prevention work in Tarragona City and may further explain both changes and differences within and between zones.

### Strengths and limitations

This study has both strengths and limitations that should be considered when interpreting the findings. One limitation of the study is that it is a repeated cross-sectional study, which means that the findings are only correlational and cannot establish causality. However, the environmental and population-level nature of the IPM does not lend itself easily to longitudinal models. Thus mixed-methods with a clear process-evaluation component are recommended. Second, only five out of the eight zones in Tarragona City were included in the study due to differences in school participation between 2015 and 2019. This should be considered when interpreting the results at the city level, as the substance use proportions may be over or under-estimated. It is possible that there were unique characteristics about the school communities that did not participate in both years, which could have influenced the results in their absence. Third, this study included a narrow age range, and questions of lifetime substance use may have been subjected to recall bias.

Regarding strengths, this study first includes a relatively large survey data set with high response rates from an area that has to date not been included in a study of this nature. Second, our analyses shed light on locale-specific findings which vary significantly between city zones and highlight the need for such approaches more generally in primary prevention.

### Implications for policy and practise

Historically, primary prevention practise has largely been limited to the implementation of educational approaches, commonly *via* instructional programs in schools (12). The theoretical assumptions of the IPM that are outlined in the Five Guiding Principles (14), and the sequential process of model execution *via* the 10 Steps to Implementation (21) emphasise a movement to an alternative focus in primary prevention which is to facilitate a greater emphasis on long-term collaboration between researchers, policy makers and practitioners *via* data-driven assessment, decision-making, public health education and intervention planning and implementation. Further, to account for environmental variation in well-established risk and protective factors and substance use outcomes which enables policy makers and practitioners to prioritise resource allocation based on locally established needs. The findings of our study confirm that the risk and protective factor domains of the IPM hold water in Tarragona, but also underline that the prevalence of both risk and protective factors and outcomes differ between zones. Future policy and practise would benefit from the utilisation of global data collection but locally distinguishable analyses and reporting to improve the accuracy of resource allocation and intervention planning based on local needs, culture, and assets.

## Data availability statement

The raw data supporting the conclusions of this article will be made available by the authors, without undue reservation.

## Ethics statement

This study is in accordance with the Declaration of Helsinki. Data was collected using passive consent to obtain a high response rate. A similar protocol is used in the ESPAD and HBSC studies. Students and parent(s)/guardian(s) were informed of the study and their rights to decline to participate at any time with no penalties. All aspects of data collection, which includes the use of passive consent, were reviewed and approved by the National Bioethics Committee of Iceland (protocol #: VSNb2017020009/04.01) and the Department of Education of the Generalitat of Catalonia in accordance with governing Data Protection and Processing of Personal Data laws of both countries.

## Author contributions

CM and AK conceived of the research question and design. IT conducted the initial cleaning of the final dataset used in the study. CM conducted analyses, prepared tables, and drafted the first manuscript. AK and IS determined the survey measures used in the study and coordinated their administration with PR. All authors provided edits and comments and read and approved the final draft.
